# Novel update of interventional strategies of vascular aging in humans

**DOI:** 10.1002/agm2.12124

**Published:** 2020-10-01

**Authors:** Yumin Qiu, Yuanya Liu, Xiaoling Liu, Jun Tao

**Affiliations:** ^1^ Department of Hypertension and Vascular Disease The First Affiliated Hospital of Sun Yat‐sen University Guangzhou China; ^2^ National Guangdong Joint Engineering Laboratory for Diagnosis and Treatment of Vascular Disease Guangzhou China; ^3^ Key Laboratory on Assisted Circulation Ministry of Health Guangzhou China

**Keywords:** interventional strategies, mechanisms, vascular aging

## Abstract

China is the country with the largest elderly population in the world. Age‐related ischemic vascular disease is on a rapidly increasing trend and has brought a huge burden on the whole society. Vascular aging, characterized by vascular dysfunction and aging of the vasculature, plays a key role in the pathogenesis of ischemic vascular disease, morbidity, and mortality of the elderly. This review describes mechanisms and depicts the novel interventional strategies of vascular aging. We propose the significance of vascular aging for early detection, early prevention, and early treatment of age‐related ischemic disease and effective improvement of the quality of life in the elderly population. Finally, future directions to develop novel interventions targeting ischemic disease are presented to prevent age‐related vascular pathologies.

## Graphical abstract

Vascular aging is characterized by vascular dysfunction and vasculature damage and plays a key role in the development of ischemic vascular disease of the elderly. Various mechanisms are involved in the pathogenesis of vascular aging and a large number of candidate interventions targeting the aging process have emerged from basic research and clinical trials, which may contribute to prevent vascular aging related ischemic disease and prolong lifespan and healthspan of the elderly.

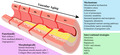

## 1 INTRODUCTION

Ischemic vascular disease is the most common cause of death among the elderly worldwide, accounting for nearly one‐third of all deaths at the age of 65 years and almost two‐thirds at the age of 85 years (World Health Organization).^1^ With the large proportion of aging population and an estimated increase of adults aged >65 years from 12.6% to 26% in the next 30 years in China, stressing age‐related ischemic vascular disease is of critical significance due to the huge economic burden and mental strain on the whole society.^2^ Vascular‐aging‐induced functional and structural alterations of vessels play a key role in the pathogenesis of ischemic vascular disease. Therefore, it is critical to elucidate mechanisms underlying vascular aging and explore the novel interventions for subclinical dysfunction and manifested disease so as to prevent the occurrence and development of ischemic vascular disease associated with old age.

## 2 MECHANISMS OF VASCULAR AGING

**FIGURE 1:**
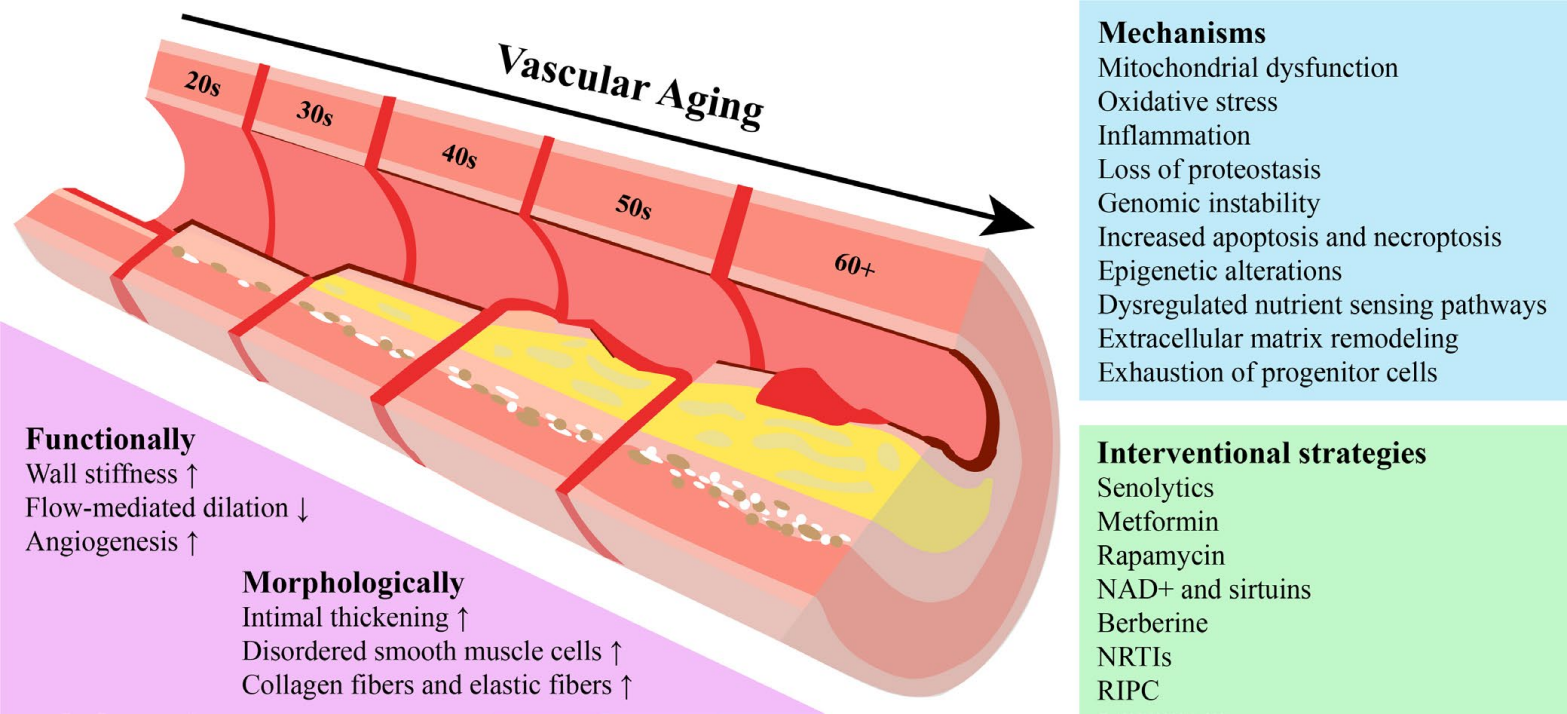
Schematic illustrations of mechanisms and interventional strategies of vascular aging in humans. NAD+, nicotinamide adenine dinucleotide; NRTIs, nucleoside reverse transcriptase inhibitors; RIPC, remote ischemic preconditioning.

Rapid advances in geriatrics have been achieved in the last 30 years and have led to an evolution on the pathogenesis of vascular aging. An increasing number of studies have demonstrated that different pathophysiological mechanisms, including mitochondrial dysfunction,^3^ oxidative stress,^4^ inflammation,^5^ loss of proteostasis,^6^ genomic instability,^7^ increased apoptosis and necroptosis,^8^, ^9^ epigenetic alterations,^10^ dysregulated nutrient‐sensing pathways,^11^ extracellular matrix remodeling,^12^ and exhaustion of progenitor cells,^13^ are attributed to the occurrence of vascular aging (Figure 1). The interaction of multiple mechanisms results in the alteration of function and structure of vessels during vascular aging.^14^

Among the above mechanisms, mitochondrial dysfunction plays a central role in the regulation of vascular aging processes.^15^ During aging, mitochondria are reduced, reactive oxygen species (ROS) production increases, a loss of function occurs in the electron transport chain, and there is a reduction in the synthesis of adenosine‐5′‐triphosphate.^16^ Recent studies suggested that in aged vasculature, the biogenesis of mitochondria was impaired^17^ and increased mitochondrial ROS contributed to loss of efficiency of electron transport chain through p66shc‐mediated oxidative stress pathways^18^ and injured nuclear factor [erythroid‐derived 2]‐like 2 (Nrf2)‐mediated antioxidant defense pathways.^19^ Treatment targeting the clearance of ROS, such as resveratrol, has been shown to attenuate oxidative stress and improve endothelial function in aging.^20^

Chronic inflammation is another hallmark of aging and age‐related inflammatory response acts as an important role in vascular dysfunction. Previous research found that there is a proinflammatory shift in the gene expression of endothelial cells, including inflammatory cytokines, chemokines, and adhesion molecules, which results in cellular metabolism disorder, increased apoptosis, vascular remolding, and finally leads to the pathogenesis of various ischemic vascular diseases.^21^, ^22^, ^23^

## 3 INTERVENTIONAL STRATEGIES OF VASCULAR AGING

Classical strategies targeting mechanisms of vascular aging to delay vascular aging and prevent disease, such as exercise, diet, and other lifestyle interventions, have far‐reaching significance. However, these alone seem to be insufficient to prevent the occurrence of geriatric disease and efforts are needed to tackle the underlying processes of vascular aging. At present, the most promising novel strategies for delaying vascular aging include improving the function of mitochondria, reducing age‐related inflammation, increasing autophagy, moderately reducing the activity of the nutrient‐sensing network, especially reducing the activity of mammalian target of rapamycin complex 1 (mTORC1), removing senescent cells, and using its own endogenous metabolites to re‐energize stem cells, and so forth (Figure 1).^24^ Several potential drugs and natural products have been reported to modulate aging.^25^, ^26^, ^27^ Shedding light on the mechanisms of vascular aging and the development of novel agents will likely reduce the risk of age‐related disease and extend the human health span.

### 3.1 Senolytics

Senolytics is a class of drugs that selectively kill senescent cells, and scientists have reported the first senolytics drug combination—dasatinib + quercetin. Recent studies demonstrated that senolytic treatment exerted a positive effect on senescent cell burden, DNA damage, vasomotor function, nitric oxide signaling, calcification, and osteogenic signaling in chronologically aged mice.^28^ Another study indicated that this combination selectively cleared senescent cells in idiopathic pulmonary fibrosis mice and improved lung function and physical health indicators in mice.^29^ In an open‐labeled phase I clinical trial, nine patients with diabetic nephropathy received dasatinib and quercetin therapy, which reduced the load of adipose tissue senescent cells.^30^ The effect of senolytic treatment may be mediated by members of the BCL‐2 family, PI3K/AKT, p53/FOXO4, HSP90, and HIF1α.^24^ These results proved that senolytics are expected to be used to delay vascular aging and prolong the life span of the elderly.

### 3.2 Metformin

Metformin is a biguanide drug widely used for type 2 diabetes.^31^ A previous study suggested that metformin increases the life span of *Caenorhabditis elegans* by up to 36%, which may be the result of AMP kinase (AMPK) activation and metabolic change of the microbiome.^32^, ^33^ A study on mice found that treatment with metformin mimics some of the benefits of calorie restriction, such as increased insulin sensitivity and reduced low‐density lipoprotein and cholesterol levels and finally improves health span and life span.^34^ The mechanisms include anti‐inflammatory, inhibiting mTOR, regulating insulin/insulin‐like growth factor 1, reducing the production of ROS, and modulating the expression of sirtuins.^35^, ^36^ Retrospective, epidemiological analyses elucidated that administration of metformin is associated with the improvement of vascular function and reductions in the incidence and mortality of ischemic disease.^37^, ^38^ The results of metformin treatment in age‐related disease are also encouraging, with a wide range of protective roles in cardiovascular disease, cerebrovascular disease, cancer, chronic kidney disease, and neurodegeneration.^37^, ^39^

### 3.3 Rapamycin

Rapamycin is a macrolide compound and was found to exert its role in immune and anti‐proliferation responses.^40^ Studies showed that rapamycin binds to FK‐506‐binding protein 12 and destabilizes and inhibits mTORC1, which is an important molecule regulating various cellular processes.^41^ Scientists discovered that inhibition of mTORC1 activity showed a favorable effect on increasing the life span and health span in different kinds of species.^42^ It was proposed that rapamycin extended the life span by up to 60% and even reversed the changes in vascular function and structure, cognition, cardiac hypertrophy, and immune senescence in aged mice, through both genetic and pharmacological modulation of mTOR signaling.^43^, ^44^ The current clinical uses of rapamycin may be limited by its adverse effect to some extent, including hyperglycemia and hyperlipidaemia.^45^ As an effective anti‐vascular aging agent, rapamycin has both advantages and disadvantages and it should be balanced for every individual.

### 3.4 Nicotinamide adenine dinucleotide and sirtuins

Nicotinamide adenine dinucleotide (NAD^+^), as a cofactor in many key biological processes of cells, is an important mediator of biochemical reactions in the body and an essential molecule in many metabolic pathways. It has been found that the concentration of NAD^+^ in human tissues gradually decreases with age, and at least decreases by 50%, accompanied by a series of pathological processes, such as chronic inflammation, oxidative stress, DNA damage, and mitochondrial dysfunction.^46^ Supplementation of NAD^+^ and its precursors is beneficial to reduce the occurrence of oxidative stress, increase the regenerative capacity of vascular endothelial cells, and prolong cell life.^24^ At the same time, sirtuins are a class of NAD^+^‐dependent deacetylases. Studies have discovered that members of the sirtuin family can reduce mitochondrial oxidative stress, promote angiogenesis, and play an important role in vascular disease, such as hypertension.^47^, ^48^

### 3.5 Berberine

Berberine is an isoquinoline alkaloid extracted from various plants, which plays an important role in lowering blood pressure,^49^ regulating blood lipids,^50^ and controlling blood glucose.^51^ It was found that berberine could activate the AMPK‐signaling pathway, and inhibit the activity of mTOR to delay cell senescence caused by DNA replication disorder, and also increase antioxidant activity by activating the NRF2‐signaling pathway to achieve the effect of longevity extension.^52^

### 3.6 Nucleoside reverse transcriptase inhibitors

Nucleoside reverse transcriptase inhibitors (NRTIs) are used in clinical HIV treatment, but can also inhibit open‐reading frame‐related reverse transcriptase activity of long dispersive elements.^24^ Recent studies have found that NRTIs, including lamivudine and stavudine, can lower the level of DNA damage and prolong the life span of Sirtuin6^−/−^ mice, and reduce senescence‐related secretory phenotypes and inflammatory responses in older mice.^53^, ^54^ These findings make NRTIs a new candidate for delaying aging.

### 3.7 Remote ischemic preconditioning

Remote ischemic preconditioning (RIPC) is a safe, noninvasive, simple, and low‐cost non‐drug device intervention and has been widely used since it was first proposed by Karin Przyklenk in 1999.^55^ RIPC is an intrinsic protective phenomenon to protect the vital organs with non‐fatal regional ischemia followed by reperfusion, through the involvement of SDF‐1α, HIF‐1α, oxidative stress, and apoptotic pathways.^56^ Short‐term RIPC treatment led to increased levels of brain‐derived neurotrophic factor and vascular endothelial growth factor in arterial plasma.^57^ A recent study has demonstrated that 1‐month RIPC treatment can significantly reduce the blood pressure of patients with mild essential hypertension and improve microvascular endothelial function.^58^ RIPC may be a novel alternative or complementary intervention means to protect against vascular aging and endothelial dysfunction.

## 4 PERSPECTIVES

All disease stems from vessels. Vascular aging is a common basis of various vascular diseases, and the normal structure and function of blood vessels are crucial for maintaining the health of the elderly. This review describes the mechanisms of vascular aging, depicts the novel interventional strategies of vascular aging, and emphasizes the important significance of vascular aging in ischemic disease. Although significant progress has been achieved in exploring age‐induced changes in vascular function and age‐related disease, further research is necessary to deeply study the molecular mechanism of vascular aging, clarify the early characteristics of vascular aging and construct a new evaluation system of vascular aging. Furthermore, preclinical studies on vascular aging and clinical trials of innovative strategies should be of high priority, which may contribute to early detection, early prevention, and early treatment of vascular‐injury‐related disease, effectively improving the quality of life and prolonging the healthy life span of the elderly.

## CONFLICTS OF INTEREST

Nothing to disclose.
